# Visual mapping of operating theatre team dynamics and communication for reflexive feedback and surgical practice optimisation

**DOI:** 10.1097/AS9.0000000000000463

**Published:** 2024-07-17

**Authors:** Surya Surendran, Candice Bonaconsa, Vrinda Nampoothiri, Oluchi Mbamalu, Anu George, Swetha Mallick, Sudheer OV, Alison Holmes, Marc Mendelson, Sanjeev Singh, Gabriel Birgand, Esmita Charani

**Affiliations:** 1 Department of Infection Control and Epidemiology, Amrita Institute of Medical Sciences, Amrita Vishwa Vidyapeetham University, Kochi, Kerala, India; 2 Health Systems and Equity, The George Institute for Global Health, New Delhi, India; 3 Division of Infectious Diseases and HIV Medicine, Department of Medicine, Groote Schuur Hospital, University of Cape Town, Cape Town, South Africa; 4 National Institute for Health Research Health Protection Research Unit in Healthcare Associated Infections and Antimicrobial Resistance, Imperial College London, UK; 5 Faculty of Life and Health Sciences, University of Liverpool; 6 Regional Center for Infection Prevention and Control, Region of Pays de la Loire, Nantes University Hospital, France

## Abstract

**Background:**

Effective operating theatre (OT) communication and teamwork are essential to optimal surgical outcomes. We mapped the OT team dynamics and infection control practices using visual methods to guide reflexive feedback and optimise perioperative practices.

**Methods:**

Data were gathered from adult gastro-intestinal surgical teams at a tertiary hospital in India using observations, sociograms (communication mapping tool) and focus group discussions (FGD). Our methods aimed to map team communication, roles and responsibilities in infection-related practices, and door openings. Qualitative data were thematically analysed. Quantitative data were analysed using descriptive statistics.

**Results:**

Data were gathered from ten surgical procedures (over 51 hours) using 16 sociograms, 15 traffic-flow maps, 3 and FGDs. Senior surgeons directly influence team hierarchies, dynamics, and communication. While the surgeons, anaesthetic residents and technicians lead most tasks during procedures, the scrub nurse acts as a mediator co-ordinating activity among role players across hierarchies. Failing to provide the scrub nurse with complete details of the planned surgery leads to multiple door openings to fetch equipment and disposables. Traffic flow observed in 15-minute intervals, corresponds to a mean frequency of 56 doors openings per hour (Min: 16; Max: 108), with implications for infection control. Implementing the WHO surgical safety checklist was inconsistent across pathways and does not match reported compliance data.

**Conclusion:**

Human factors research is important in optimising surgical teamwork. Using visual methods to provide feedback to perioperative teams on their communication patterns and behaviours, provided opportunity for contextualised enhancement of infection prevention and control practices.

## Abbreviations

AMS: Antimicrobial stewardship
FGD: Focus group discussions
GI: Gastro-intestinal
HCWs: Healthcare workers
IPC: Infection prevention and control
OT: Operating theatre

## Introduction

The operating theatre (OT) is a closed clinical environment where professionals from multiple disciplines and with various training, are required to work in a closely coordinated manner. Despite significant improvement in safety, errors routinely occur in surgical care, including in the OT. Around 14% of patients suffer an adverse event during an episode of surgical care with 40% of these events deemed preventable.^1^ Among these, post-operative infections are the most common complications in surgery, with the highest incidence rates reported after open abdominal procedures.^2,3^

The complex OT environment provides multiple opportunities for errors, not only from technical incompetence but also from poor interpersonal skills. Disruptions in the flow of an operation, due to teamwork and communication failures, significantly contribute to such adverse events.^4^ Current efforts for the prevention of post-operative infections focus on technical measures, with less attention paid to teamwork and communication in the OT to improve uptake of preventive measures.^5^ Non-technical skills for surgeons including situation awareness, decision-making, communication and teamwork, and leadership are key to the prevention of adverse events, including infection across the surgical pathway.

Interprofessional collaboration in surgical teams has been widely studied due to the impact of human factors on patient safety. Professional power, hierarchy, patriarchy and gender norms have been described as factors influencing interprofessional collaboration, creating barriers to communication between surgeons and nurses.^6–8^ Currently, despite evidence suggesting a strong element of interprofessional teamwork in the OT, the roles and responsibilities between OT team members seem ill-defined.^8,9^ The OT also presents opportunities for effective leadership to generate a supportive environment and better use of resources.^7,8^ Communication skills are the vehicle to establish effective teamworking.^10^ Teams should have a shared understanding of the current situation, immediate and upcoming tasks, and the ultimate goal.^4,11^ Patterns of communication in the OT are complex, socially motivated and are usually setting specific.^12–14^ Common team communication failures in the OT demonstrate visible effects on system processes such as inefficiency, team tension, resource waste and delay, and patient inconvenience.^15^ Distractions (such as noise, traffic flow) may also compromise performance.^16,17^ Previous studies have found that more distractions and higher noise levels are related to poorer teamwork in the OT,^18^ and that more lapses in discipline (operationalised as traffic, noise and visitors) are related to a higher incidence of post-operative complications,^19^ such as surgical site infections.^20^ Moreover, door opening affects efficacy of the ventilation system and increases the risk of post-operative infections and complications.^21–23^

The implementation of methods or techniques developed to structure and optimise communication, and improve surgical teamwork^24^ often encounters multiple barriers. For example, gaps remain in compliance and engagement with the WHO surgical safety checklist^25^ despite a flexible design and context-fit implementation.^26^ Monitoring and feedback on teamwork and communication is critical for effective and continuous quality improvement of non-technical skills in the OT.^27^ We investigated how ethnographic research can be used to map the OT team dynamic with a focus on infection related practices to aid reflexive visual feedback to teams.

## Methods

### Study design

The work formed a part of the multisite study where we applied ethnographic observations to identify contextual factors that influenced antibiotic decision making and infection management practices across surgical pathways.^28–33^ The novel application of sociograms to capture ward round communication on infection care was developed during the project at one site ^30^ and was then transferred to the second site. We identified an opportunity to work with surgical teams and investigate team dynamics in the OT. We used three methods to map and describe teamwork and communication related to infection prevention and control (IPC) practices during surgical procedures: (i) non-participant observations using a data collection guide, developed through a review of the literature and previous work of the research team^29,34^, (ii) sociograms to map IPC focused communication patterns and team dynamics in the OT, and, (iii) counts of door openings to capture traffic flow. Focus group discussions (FGD) with OT participants were conducted for reflexive feedback discussions, using the data gathered. A researcher with social science background (SS) and an understanding of surgical settings, IPC and AMS practices led the data collection, analysis, and feedback.

### Setting

Between Nov 2021 to February 2022, data were gathered from observations in the gastro-intestinal (GI) surgical department of a 1350 bedded not-for-profit, tertiary hospital in south India. This specialty was chosen due to high turnover of patients, variable operating procedure times, and high infection risk surgical procedures.

### Data collection

All healthcare workers (HCWs) directly or indirectly involved in the surgical care of the patient, entering the OT at the time of observation, were included in the study. Prior to the data collection, consent was obtained from the GI chief surgeon and the head of anaesthesia and the nurse in charge. Verbal consent was obtained from HCWs present in the OT on days of data collection after informing them about the study and broad objectives of the researcher’s presence.

**Figure 1:**
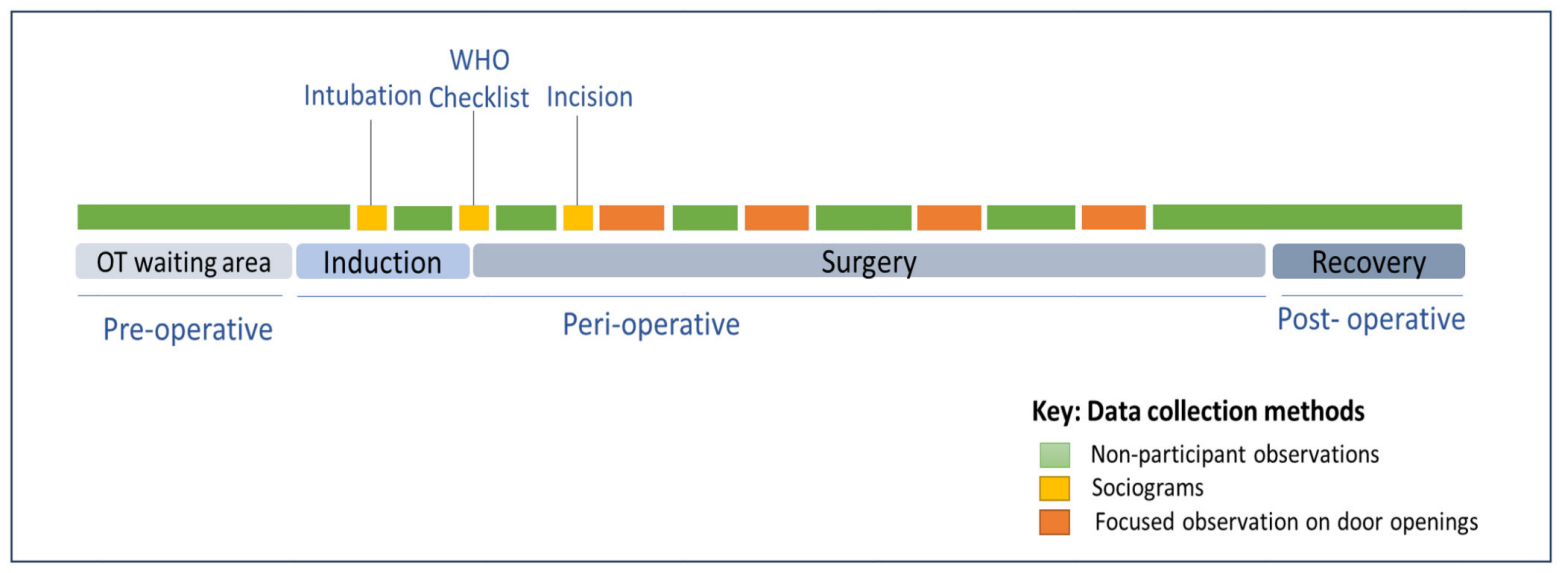
Illustration of data collection methods along the patient’s surgical pathway

Ethnographic observations captured all clinical activities and communication between role players (including the patient) along a timeline from patient in the pre-operative room to the post-operative recovery room (Figure 1). Roles played by each HCW regarding pre-operative (communication with patient/carer, intubation, surgical antibiotic prophylaxis, catheter insertions, patient positioning and draping), intraoperative (main surgical tasks including surgical incision and closure, availability of supplies, monitoring of parameters), and postoperative activities (extubations, communication with patient/carer, cleaning) were observed.

Team communications regarding IPC-related interactions were captured using sociograms on a pre-validated data collection sheet, based on existing research carried out by the team.^30^ We focussed on three specific components: (i) team interactions during patient intubation which included insertion of central lines and Foley’s urinary catheters, (ii) the surgical incision, and (iii) the WHO checklist discussion. Counts of door openings to capture traffic flow were performed in 15-minute episodes every hour from the time of surgical incision until wound closure using a validated tool to guide data collection.^35^ Time in minutes and hours was used as a reference to relay events. Details on the role player and the reason for movement were documented through observed actions or communication. Non-identifiable reasons for doors opening were categorised as ‘unknown’.

Ethnographic observations were conducted across four surgical procedures to generate a contextual understanding of the current team practices. Door openings and mapping of sociograms commenced from the fourth and fifth observations respectively. The result was a structured and standardized data collection process. We used convenience sampling and collected ethnographic data until saturation was reached and no new themes emerged. Figure 1 illustrates the data collection plan and application of methods along the patient’s surgical pathway.

Thereafter, FGDs were organised with members of the OT teams for reflexive feedback. The main purpose was to provide teams with visual data on their practices and facilitate a discussion to gain their thoughts and insights. A discussion guide, drawn up by the researchers, aimed to elicit information on the rationale to practice that was observed in the OT. Recordings from FGDs were transcribed and reviewed.

### Data analysis

Iterative data analysis, drawing on elements of grounded theory,^36^ explored themes and relationships within the data collected, aided by NVivo V.12 software and commenced shortly after the ethnographic data collection started. Two researchers (SS and CB) began by each independently coding the same subset of data and comparing the coding, and through regular discussion and consensus, developed a draft framework of the emerging themes. This framework was then used to code the remaining data. Constant comparison enabled within-narrative and between-narrative comparison of the emerging data and the identification of key themes.^37,38^ Cross-cutting themes relevant to communication on IPC were identified within the framework. The themes identified through the qualitative framework allowed for contextual analysis of the sociogram data. Sociograms were interrogated to extract the topic of individual interactions initiated by OT staff members. In preparation for the FGDs, data analysis was completed two weeks after the last patient pathway observation.

### Ethical Approval

Ethical approval for the study was granted by the Institutional Ethics Committee at Amrita Institute of Medical Sciences (IEC-AIMS-2018-INF.CONT-005A).

## Results

We conducted over 51 hours of direct observations in the OT across ten surgical procedures (Table 1). Sixteen sociograms were generated to map team communication regarding IPC-related interactions in seven surgical procedures. The team dynamic through intraoperative door openings in the operating room was assessed during fifteen-minute time points across seven surgical procedures. Finally, three focus group discussions were held with surgeons and surgical residents (6 participants); anesthetists and anaesthesia technicians (3 participants); and nurses and surgical technicians (7 participants).

### Roles, tasks and practice domains within the OT teams

Surgical care of the patient requires an overlap of various activities by multiple role players across surgical, anaesthesia and nursing teams. Table 2 maps the explicit roles and responsibilities of various HCWs as identified by the observations. HCWs share an understanding about roles, associated tasks and domains of practice which are identified through repeated observed routine activities. The co-ordination of specific surgical tasks is usually activated through a verbal directive.

Shifts in leadership and communication on the direction of care are noted between teams throughout surgical procedures. Nurses and surgical technicians prepare the OT for the surgical procedure. The anesthesia team lead the preparatory phase which includes intubation. After intubation, the command shifts to the surgical residents. As a norm, the senior surgeons enter the OT after the surgical incision. After surgical closure and during extubation, the anesthetist resumes leadership of the surgical process. Nurses and technicians are present throughout and the scrub nurse maintains a pivotal role in directing activities requested by the surgeon or anesthetist.

During the feedback discussion, team members concurred with the role allocations observed. The surgeons explained that the communication pathway was intentionally designed to keep/maintain focus on the patient undergoing surgery. (X1,Table 3).

### Team dynamics and flow of communication in the OT

**Figure 2:**
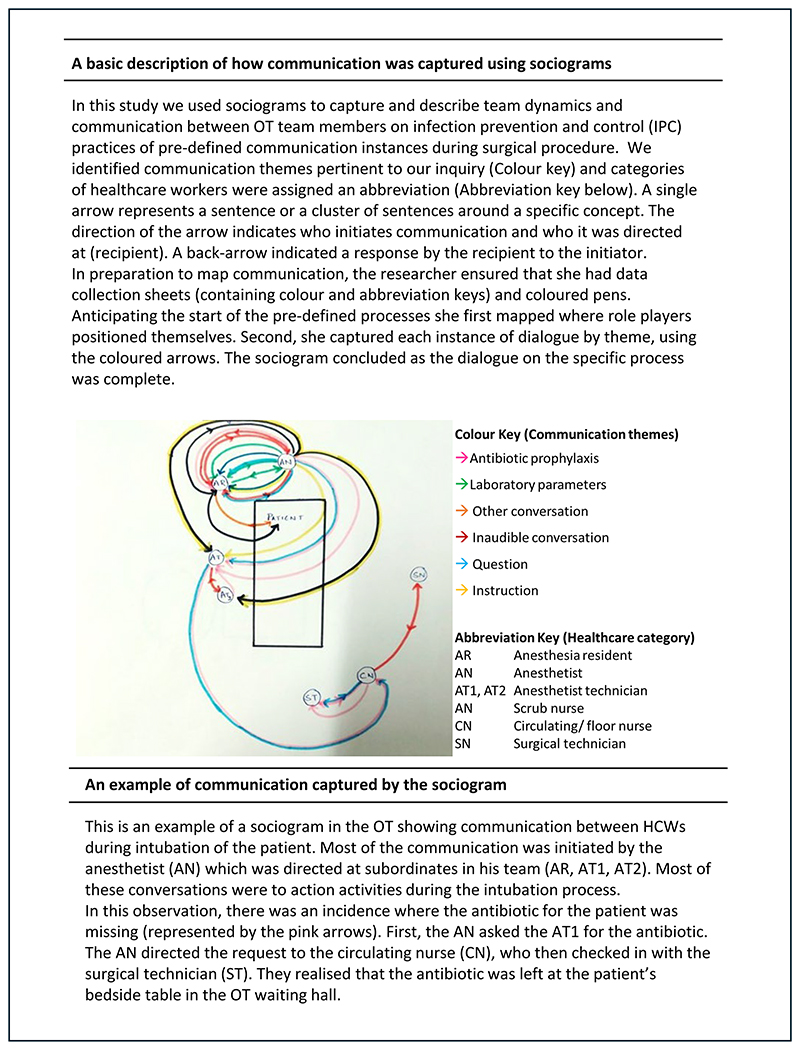
Sociogram illustrating the communication during a patient intubation

Communication is generally direct and between role players in the OT. Surgeons and anesthetists communicate clear requests which are directed at specific role players (X2, Table 3). A delay in response is noted where requests or instructions are not directed at specific role players. The scrub nurse who assists the surgeon in the procedure, acts as a mediator for the flow of communication between the surgical team and the supporting staff in the OT. Requests or instructions to arrange materials are directed at the scrub nurse, who in turn, re-directs these to the surgical technician or the circulating nurse. Initiation of communication is usually top-down where nurses and technicians mostly respond to communication initiated by surgeons and anesthetists. Positive team dynamics are noted by support staffs’ response to a non-verbal request for equipment, such as an ‘out-stretched palm’ or ‘pointed with their eyes’. Figure 2 illustrates communication patterns through a sociogram, alongside a description.

Verbal communication during routine tasks, such as administering antibiotic prophylaxis and inserting invasive devices, was minimal, posing a challenge in mapping the sociograms. Sociograms did however aid in identifying HCWs involved in the procedures and the communication direction during these procedures, even when the dialogue was not necessarily specific to infection care. Before the procedure, the senior surgeon communicated the required antibiotic choice with the resident. Typically, the resident announced the first dose administration, but not the second. At the procedure’s end, the circulating nurse asked the resident for the timing of the second administration to complete details on the anaesthetic records.

The presence of a senior surgeon alters the patterns and the ease of HCW communication in the OT. In their absence, the flow of communication is casual and open. In contrast, when the senior surgeon is present, the room is quieter and spontaneous conversations between HCWs are less likely. In their absence, the scrub nurse plays a lead role in supporting the surgical resident (X3, Table 3).

The scrub nurse ensures that sufficient equipment and consumables are available for planned and unplanned patient-specific surgical needs. However, they are not always informed on the next steps in surgery in a timely manner. While routine procedures and their experience enable some level of planning, instances are noted where they receive late unanticipated instructions. This impacts the ready availability of required materials and instruments. Last-minute runs to procure required items delay and slow down the surgical process (X4, Table 3).

### Uptake and application of the WHO safety checklist

**Figure 3:**
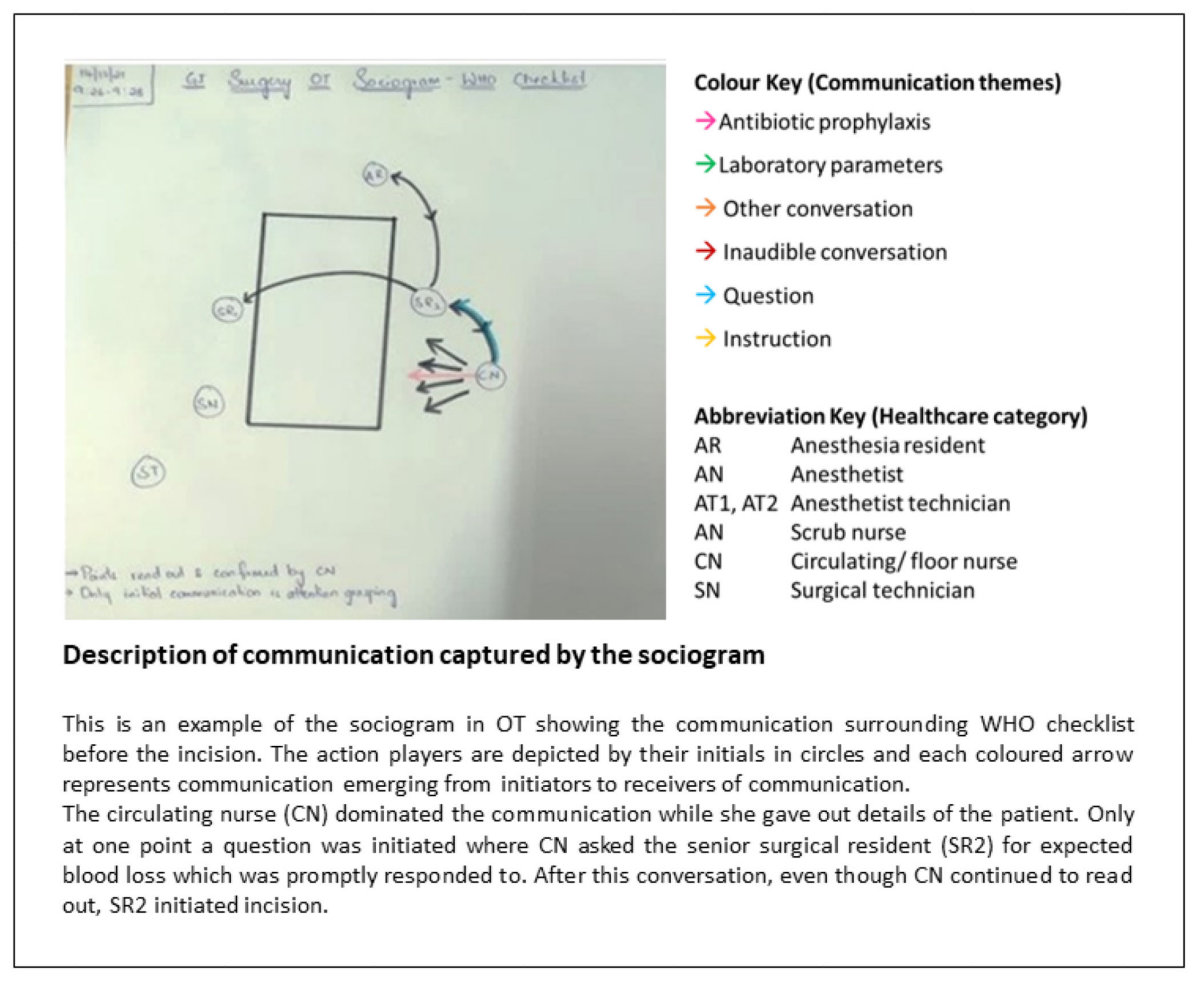
Communication surrounding WHO checklist before surgical incision

While completing the WHO checklist ^25^ during the patient’s surgery is a requirement, it is rarely discussed in practice. The checklist is referred to in three out of the ten observed surgical procedures (X5, Table 3). Within this small sample, practices to complete the checklist varied. In one observation, the form was completed in silence with no verbal check-in from the team members, while in another, the circulating nurse read out the various sections. Figure 3 illustrates a WHO checklist check in, led by the circulating nurse.

The sociogram data on WHO checklist communication were presented to the HCWs during the feedback meetings to gauge their response to the inconsistent checklist utilisation. While expressing surprise, the participants linked the poor compliance to a lack in nurse confidence. (X6, Table 3) The high turnover of nursing staff was also referred as a contributing factor especially when newly joined nurses were tasked to lead the check-in. A strategy suggested during the FGD was to empower the nurses.

### Traffic Flow in the OT environment and their reasons

**Figure 4:**
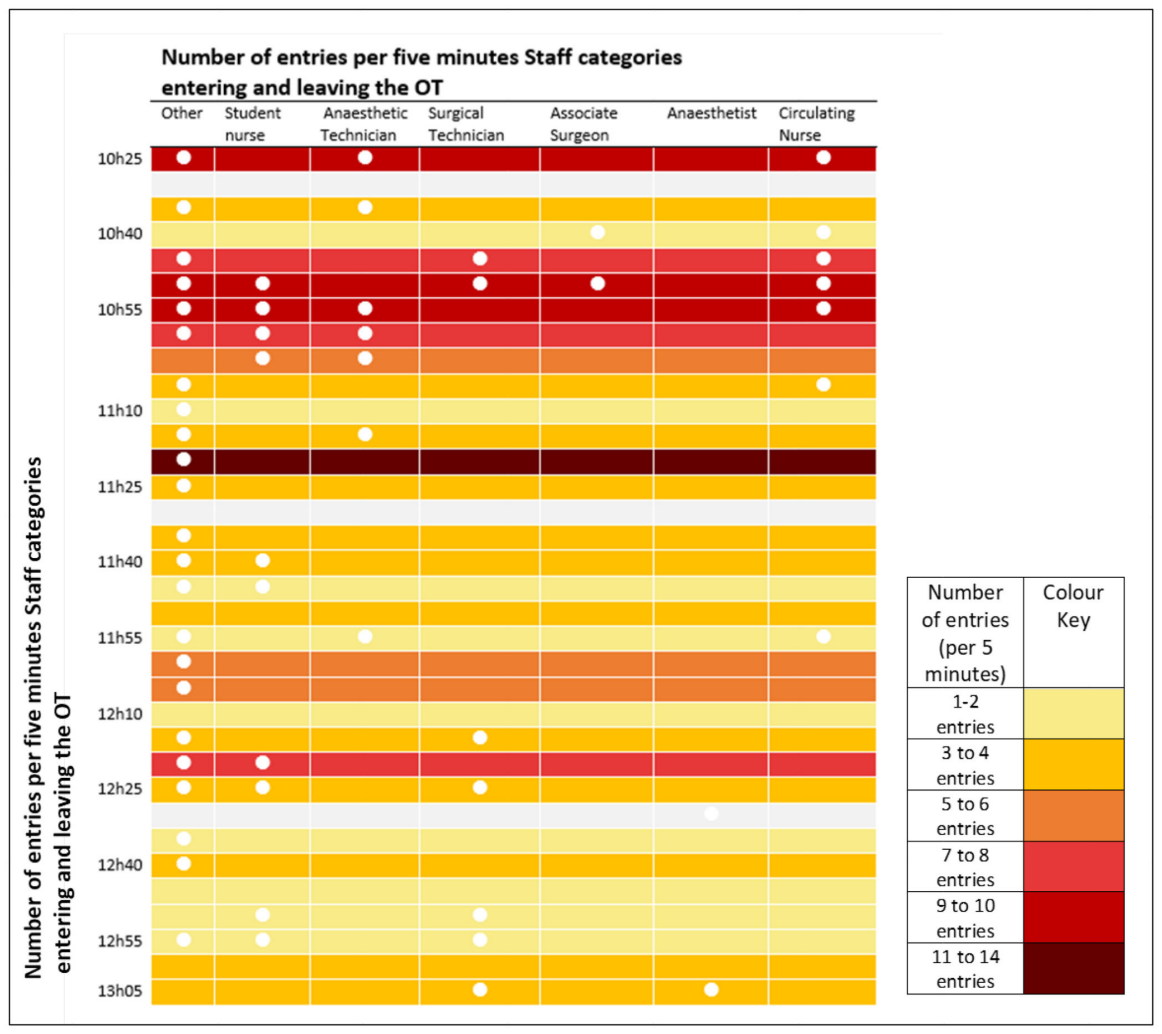
Illustration of the number of door openings per five minutes and categories of staff identified in the traffic flow from incision (10h25) to closure (13h05) from one bed space observation.

Among the 15 observations of staff traffic flow captured across seven surgical procedures, a mean of 14 door openings in a 15-minute slot is observed, corresponding to an average frequency of 56 door openings per hour. The frequency varies between 19.2 and 52.8 door openings per hour, with a range between 4 and 27 door openings. During these transits, sometimes, the door is unintentionally left half open. Doors are mostly opened by the outside staff (42.1%) followed by surgical technicians (19.1%) and nurses (16.3%). Figure 4 provides a visual illustration of the frequency of door openings in one procedure from incision to closure and demonstrates staff movement (by category) in five-minute time intervals.

Figure 4 was presented to the OT teams to provide an opportunity to reflect on and analyse their own practices. OT members explained that senior surgeons made decisions on specific surgical equipment or supplies. Since they generally only arrived after incision, there was a delay in procuring what was needed, often resulting in increased traffic flow and further door openings (X7, Table 3).

The surgical and nursing team considered introducing a checklist of required items for each procedure to ensure timely procurement ahead of the surgery.

HCWs or others who were not involved in the patient’s surgery largely contributed to the flow of traffic through the theatre. A lack of central storage meant that surgical equipment and supplies were kept in cupboards across various OTs causing traffic flow (X8, Table 3). Additionally, intra-operative replacement staff to enable short relief breaks during longer procedures or, informal check-ins with colleagues and patients in neighbouring OTs, generally for matters unrelated to the patient, also contributed to frequent door openings (X9, Table 3).

On reflection, the anesthesia team proposed a dedicated storage space closer to the OTs and the surgical team recommended adaptions to the design of a new OT that was underway. The adaption was to change the position of the bed to reduce the disturbance in airflow caused by door openings. Since most of the anaesthetist’s work was concentrated at the head-end of the bed, having the head of the bed closest to the door, meant that traffic flow for anesthesia related equipment was reduced through the rest of the theatre.

## Discussion

In this study, we used innovative tools to investigate team dynamics and practices in the OT to understand its influence on IPC outcomes, and to provide reflexive feedback opportunity to surgical team members. Data methods employed highlighted (i) the strong influence of the senior surgeon in the team hierarchies, dynamics and communication, (ii) the place of non-verbal communication in routine activities, (iii) the role of the scrub nurse as a mediator, coordinating tasks/activities across hierarchies, (iv) the lack of anticipation and communication on plan of events generating a high traffic flow, (v) the poor communication around the WHO checklist. The presentation of these results via visual methods provided reflexive feedback discussion leading to actions for improvement such as nurse empowerment, proposal for a new storage room for anaesthesia equipment and change in design of the OT structure itself.

The role played by HCWs in the operation theatre is critical for the successful outcome of surgical procedures.^39^ Role demarcation across staff members was previously considered to be one of the elements of ‘good’ teamwork, with team members’ ability to effectively fulfil their separate roles contributing to efficient team functioning.^8^ During the surgical procedure observed in the present study, individual roles, associated tasks and domains of IPC practice on routine activities were shared between role players. Team dynamics in the OT was altered by the presence of the senior surgeon with changes observed in the level of spontaneity between all categories of HCW communication and initiators of communication. The influence of hierarchy on team dynamics has been reported by several other studies.^6,40–42^ In a qualitative study from Sri Lanka, professional power, hierarchy, socialization, patriarchy and gender norms influenced interprofessional collaboration and created barriers to communication between surgeons and nurses.^8^ As junior surgeons derive their understanding of appropriate practices mainly from observing senior surgeons, the development of non-technical skills by senior surgeons through reflexive feedback of observations on that domain appears critical to improve communication in future generations. The identification of key stakeholders may lead to targeted interventions to improve communication and promote patient safety. By providing targeted training, organisations can ensure that their staff members are equipped with the necessary knowledge and skills to carry out their roles effectively.

Communication plays an important and complex role in the operating theatre. Without effective communication to create inclusive environments, and coordinate the multiplicity of tasks involved in surgery, teamwork cannot be successful. An ethnographic study conducted in Denmark described the silent and ordinary communication pattern as guided by shared goals and characterized by mutual respect, among teams performing routine surgical procedures of short duration.^14^ In our study, non-verbal communication highlighted responsive teamwork and the experience of working together. However, in some cases, such as teams with large turnover, non-verbal communication may lead to breaches in the multiplicity of tasks involved in surgery and unsuccessful teamwork. As performed in the present study, the use of non-verbal communication can be examined by direct observations of work practices in the OT,^10^ for reflexive feedback on the efficacy and ways to improve clinical practice and feed into training and education.

A qualitative evaluation of the WHO checklist described how utilisation is influenced by a range of organisational, system, team and checklist-specific factors.^43^ A lack of buy-in and insight into the relevance of the tool (which was also noted in our study) and staff feeling that it is a time-consuming exercise creating inefficiencies in the running of operating lists are some of the reasons for suboptimal use.^43^ Identifying setting specific challenges requires locally collected data that is personally relevant and persuasive to local teams. A gap remains in developing methods by which the impact of the checklist on case-by-case team performance can be captured and fed back to teams.^43^

The traffic flow may appear as a surrogate of the discipline and teamwork in the OT but also of the infectious risk and the overall patient safety. Among the 10 observations, the frequency varied between 19.2 and 52.8 per hour while the analysis of published studies described a frequency varying from 5 to 102/h ^27^. The main documented reasons for door openings were a need for supplies or information, but the reason remains unknown in half of these openings. Door openings adversely affect air exchange, air quality, and positive pressure in the OR compared with adjacent rooms,^44^ but also increase interruptions and distractions.^19,45^ As found in the present study, the unnecessary entries/exits, estimated in the literature to be approximately 60% of the total, are mainly due to a lack of preparation and organisation. Several easy elements could lead to improvement: the storage of components and frequently used instruments in the OR, a clear and advanced communication strategy, a shift change of the surgical team prepared in advance, a sign on the door advising caution, proper education of OR team and visitors, and a robust audit process. The leadership of the surgeon and the head nurse is probably the cornerstone of the discipline and the organisation in the OR. The awareness and improvement process through regular audit and feedback may lead to decreased risks associated with the traffic flow during procedures.

We found the use of visual methods for feedback to be an effective means of promoting team reflection and enhancing awareness of practices among surgical teams.^30^ This approach facilitates the processing of complex information by presenting it in a visually engaging and easy-to-understand format.^46^ By using visual aids, such as graphs, charts, and diagrams, surgical teams are better able to comprehend the data and identify areas that require attention. This was also found in a study that used visual tools for flow mapping in the OR.^47^ The visual feedback approach encourages team collaboration and communication, allowing team members to discuss and analyse the information together. Overall, the use of visual methods for feedback has proven to be an efficient tool for promoting self-reflection and improvement within surgical teams, ultimately contributing to the delivery of high-quality patient care.^30,48^ Utilising multiple visual tools to aid reflexive feedback to teams can be readily implemented by hospitals to enhance teamwork and communication among HCWs. In addition to providing specific recommendations to the study setting, supplemental table 1 provides recommendations for how other settings could use similar methodologies for quality improvement to map and improve OT theatre team dynamics and communication on AMS and IPC-related practices.

This study has several limitations. First, since there was no standard method in place for such a study, we leveraged our prior experience with data collection at the study site to formulate a data collection strategy. During the initial stages of the study, this approach necessitated a trial-and-error process to refine and standardise our methodology. Second, observations were conducted in one surgical speciality in a single centre during surgeries of relatively shorter duration. The findings may not provide a comprehensive understanding of OT practices, which are likely to vary across different surgeries. Third, HCWs were aware of the observations as we asked for their consent, potentially leading to a Hawthorne effect. Fourth, we did not factor the gender of the team members in the analysis and interpretation of our results. Gendered hierarchies in surgical teams may influence team dynamics and communication. Finally, whilst our study revealed significant interest among HCWs, the long-term impact and sustainability of these changes could not be ascertained due to the limited duration of our investigation.

In conclusion, the feedback from ethnographic observations through visual representations and FGDs represents an opportunity to routinely improve human factors in the operating room. In this study, this strategy highlighted the influence and roles of stakeholders on the communication, teamwork and traffic flow guiding meaningful feedback and accurate actions to enhance practices and ensure safe patient outcomes in gastro-intestinal surgery.

## Supplementary Material

Supplemental Data File

## Figures and Tables

**Table 1:** Characteristics of patients, surgical procedure and OT included in the study.

Surgical case number	Patient details (age/ gender)	Surgery	Duration of observation	Number of focused observations to tally OT door openings	Number of sociograms
1	60/Female	Transverse colectomy	6 hrs 37 mins	0	0
2	63/Male	Sigmoid colectomy	6 hrs 46 mins	0	0
3	55/Female	Laparoscopic hernia repair with mesh	2 hrs 50 mins	0	0
4	50/Female	Laparoscopic hernia repair with mesh	3 hrs 16 mins	2	0
5	51/Male	Hernia repair with mesh (open)	5 hrs 12 mins	3	2
6	59/Male	Laparoscopic cholecystectomy	3 hrs 50 mins	3	4
7	66/Female	Diagnostic laparoscopy sleeve resection of gastrointestinal stromal tumor	3 hrs 38 mins	2	2
8	49/Female	Trans anal endoscopic operation	3 hrs 17 mins	1	3
9	66/Male	Laparoscopic cholecystectomy	2 hrs 25 mins	2	2
10	80/Male	Diagnostic laparoscopy and partial colectomy	3 hrs 00 mins	2	3

OT, operating theatre

**Table 2:** Roles and responsibilities of different stakeholders, as identified during observations (▲ primary role, ◯ secondary role)

Activities		Senior surgeon	Surgical Resident	Surgical Technician	Anesthetist	Anesthesia resident	Anesthesia technician	Scrub nurse	Circulating nurse
**Pre-operative**	Communication with patient/carer			**▲**	**○**	**▲**	**○**		**○**
	Intubation				**○**	**▲**	**▲**		
	Administering antibiotic prophylaxis				**○**	**▲**	**▲**		
	Foley’s insertion		**▲**	**○**					
	Intravenous line insertion				**○**	**▲**	**▲**		
	Central line insertion				**○**	**▲**	**▲**		
	Patient Positioning		**▲**	**▲**		**○**	**○**		**○**
	Draping patient		**▲**	**○**			**○**	**▲**	
**Peri-operative**	Wound incision		**▲**						
	Assuring availability of surgical instruments in OT			**▲**			**○**	**▲**	**▲**
	Performing the surgery	**▲**	**▲**						
	Surgical Assistant		**▲**					**▲**	
	Monitoring patient parameters					**▲**			
	Wound closure		**▲**						
**WHO Checklist**									**▲**
**Documentation-on board, papers/forms**			**○**			**▲**			**▲**
**Post-operative**	Extubation			**○**	**○**	**▲**	**▲**		**○**
	Communication with patient/carer			**○**	**○**	**▲**	**○**		
	Cleaning the OT			**○**			**○**	**○**	**○**

**Table 3:** Illustrative excerpts from field notes and focus group discussions to support the key emerging themes

Theme	ID	Quote
Roles, tasks and practice domains within the OT teams	X1	“That is how the system is designed. The scrub nurse is the point of communication for all things. The surgeon is supposed to be only focused on the field. He is not supposed to be trying to talk to the circulating [nurse]. The scrub nurse’s job is the point of communication and like our team manager, kind of all instruments, all materials, organising...” Focus group discussion, Surgeon.
Team dynamics and flow of communication in the OT	X2	The surgical resident said, “Get ready for local anesthesia.” The anesthetic registrar filled a syringe and gave it to the scrub nurse. Observation notes, Observation number 4.
	X3	The needle fell off while the surgical resident was trying to make the stitch inside the wound. The scrub nurse said “Don’t hold so firmly. Hold it gently”. The resident nodded and tried again.’ Observation notes, Observation number 3
	X4	Surgeons left the OT mid procedure to attend to something else and the nurses and technicians were having a conversation. The scrub nurse said, “Don’t know what they will be doing next and how much more time they will take...” Observation notes, Observation number 2
Uptake and application of the WHO safety checklist	X5	While the surgery was underway, I saw the anesthetic resident signing some forms. One of the forms included the WHO checklist. Except for the completion of the patient demographics, the rest of the form was blank. The checklist was not discussed today. Observation notes, Observation number 7
	X6	“I think sometimes the circulating nurse is the junior person and at times when they start it (WHO Checklist) at the very beginning of the surgery, anesthetists are hectic, surgeons are hectic and they are a little scared to tell everybody to stop, finish the checklist, and then proceed. So, that is not happening, so everybody is overriding this one. I have seen it a few times including when I was operating. They will start and we will be opening like you said” Focus group discussion, Surgeon
Traffic Flow in the OT environment and their reasons	X7	“We only have one mesh for hernia, and we found out that it is not kept in the OT, also things were taken and misplaced, and items were charged to certain staff or just charged to the patient unnecessarily. Those kinds of issues have made the system such that certain items are only fetched when they are asked for during the operation.” Focus group discussion, Surgeon
	X8	“We do not have a storeroom- a common place to keep, what we do have, you know, all the serial forms are kept in OT 11. It is like that. The glucometer is kept in OT 14. Anybody needs from that corner will come to that OT” Focus group discussion, Anesthetist
	X9	“Each time, you know, somebody has to come in to relieve. Because our days are long. So, they need to be relieved to go the toilet, to go for a cup of coffee or you know those things, someone has to come in for that.” Focus group discussion, Anesthetist

OT, Operating theatre
