# Supplementary material for: Visual Mapping of Operating Theater Team Dynamics and Communication for Reflexive Feedback and Surgical Practice Optimization
Source: Ann Surg Open. 2024 Jul 17;5(3):e463. doi: 10.1097/AS9.0000000000000463 (PMC11415113; doi:10.1097/AS9.0000000000000463)
Supplement: Supplementary file 1 [file as9-5-e463-s001.pdf]

Objectives	General recommendations	Specific recommendation to local study site based on findings
Map and review operating theatre (OT) team dynamics and communication on antimicrobial stewardship (AMS)and infection prevention and control (IPC) related practices	Collect data quarterly and discuss emerging patterns through reflexive OT team discussions.<br>Data collection methods can include:<br>**Observations:** to describe roles played by each healthcare worker (HCW) regarding pre-operative (communication with patient/carer, intubation, surgical antibiotic prophylaxis, catheter insertions, patient positioning and draping), intraoperative (main surgical tasks including surgical incision and closure, availability of supplies, monitoring of parameters), and postoperative activities (extubation, communication with patient/carer, cleaning)<br>**Mapping communication and care practices:** Based on preliminary observations choose key points to capture communications in the OT. Based on our findings, it could be i) team interactions during patient intubation which included insertion of central lines and Foley`s urinary catheters, (ii) the surgical incision, and (iii) the WHO checklist discussion.<br>**Counts of door openings** implement a mechanism for standardized frequency of door opening monitoring.	Collect data quarterly and discuss emerging patterns through reflexive OT team discussions.
Optimise team dynamics and communication practices to ensure effective AMS and IPC	Explore current roles and responsibilities in OT-related AMS and IPC.<br>Discuss findings from observations and sociograms on team verbal and non-verbal communication practices.	Routinely collect data on antibiotic prophylaxis administration for entire duration of surgery to capture if additional doses are administered.
Optimise the application of the WHO checklist	Examine the teams' perspectives on the use and feasibility of the WHO checklist and provide feedback on its correct use.<br>Identify current practices and role players involved in completing the WHO checklist.<br>Identify gaps that hinder optimal checklist use.<br>Explore possible solutions with the teams.<br>Provide capacity strengthening e.g. training for active role players to enable them to complete the WHO checklist correctly.	Provide training to optimise the nurse's role in leading the discussion on WHO checklist completion.<br>Ensure that all activities are paused during the WHO checklist and that it is fully completed for each procedure.<br>Encourage active participation from all team members during the WHO checklist completion. Each stakeholder should attentively respond to questions posed by the circulating nurse.
Minimise traffic flow: regular audit and feedback may lead to better understanding of the reasons for OT interruptions and door openings leading to decreasing unnecessary traffic flow during procedures.	Identify the frequency of door openings across teams and for different procedures.<br>Identify system-related causes/ reasons for door openings.<br>Discuss with teams regarding the reasons and probable solutions to minimise door openings and strategize their implementation.	Communicate strategies and tasks to nurses and surgical technicians well in advance to facilitate preparedness and coordination among team members, minimising delays and door openings.<br>Introduce a checklist of required items for each procedure to ensure timely procurement.<br>Allocate a specific area within the OT department as central storage for anaesthetic equipment.<br>Implement a strict infection prevention strategy by restricting unnecessary walk-ins by staff not involved in the surgical team for patient on the operating table.

Supplemental Table 1: Recommendations to map and improve operating theatre (OT) team dynamics and communication on antimicrobial stewardship (AMS) and infection prevention and control (IPC) related practices through team reflexivity
